# A Mobile-Based App (MyChoices) to Increase Uptake of HIV Testing and Pre-Exposure Prophylaxis by Young Men Who Have Sex With Men: Protocol for a Pilot Randomized Controlled Trial

**DOI:** 10.2196/10694

**Published:** 2019-01-07

**Authors:** Katie B Biello, Elliot Marrow, Matthew J Mimiaga, Patrick Sullivan, Lisa Hightow-Weidman, Kenneth H Mayer

**Affiliations:** 1 Center for Health Equity Research Brown University School of Public Health Providence, RI United States; 2 Department of Behavioral & Social Sciences Brown University School of Public Health Providence, RI United States; 3 The Fenway Institute Fenway Health Boston, MA United States; 4 Department of Epidemiology Brown University School of Public Health Providence, RI United States; 5 Department of Epidemiology Rollins School of Public Health Emory University Atlanta, GA United States; 6 Behavior and Technology Lab Institute for Global Health and Infectious Diseases University of North Carolina at Chapel Hill Chapel Hill, NC United States; 7 Division of Infectious Diseases Beth Israel Deaconess Medical Center Boston, MA United States; 8 Department of Global Health and Population Harvard TH Chan School of Public Health Boston, MA United States

**Keywords:** adolescents, HIV, men who have sex with men, mHealth, mobile phone, pre-exposure prophylaxis

## Abstract

**Background:**

HIV incidence is growing most rapidly in the United States among young men who have sex with men (YMSM). Overwhelming evidence demonstrates that routine testing and expanded use of pre-exposure prophylaxis (PrEP) would dramatically reduce the population burden of HIV; however, uptake of both interventions is suboptimal among young adults. The use of mobile phone apps by YMSM is ubiquitous and may offer unique opportunities for public health interventions. MyChoices is a theory-driven app to increase HIV testing and PrEP uptake. It was developed by an interdisciplinary team based on feedback from a diverse sample of YMSM.

**Objective:**

The aim of this paper is to describe the protocol for the refinement, beta testing, and pilot randomized controlled trial (RCT) to examine the acceptability and feasibility of the MyChoices app.

**Methods:**

This 3-phase study includes 4 theater testing groups for app refinement with a total of approximately 30 YMSM; for beta testing, including quantitative assessments and exit interviews, with approximately 15 YMSM over a 2-month period; and for a pilot RCT with 60 YMSM. The pilot will assess feasibility, acceptability, and preliminary efficacy of the MyChoices app, compared with referrals only, in increasing HIV testing and PrEP uptake. All participants will be recruited at iTech clinical research sites in Boston, MA, and Bronx, NY.

**Results:**

App refinement is underway. Enrollment for the pilot RCT began in October 2018.

**Conclusions:**

MyChoices is one of the first comprehensive, theory-driven HIV prevention apps designed specifically for YMSM. If MyChoices demonstrates acceptability and feasibility in this pilot RCT, a multicity, 3-arm randomized controlled efficacy trial of this app and another youth-optimized app (LYNX) versus standard of care is planned within iTech. If shown to be efficacious, the app will be scalable, with the ability to reach YMSM across the United States as well as be geographically individualized, with app content integrated with local prevention and testing activities.

**International Registered Report Identifier (IRRID):**

PRR1-10.2196/10694

## Introduction

### Background

In the United States, HIV incidence is growing most rapidly among young men who have sex with men (YMSM). More than 20% of all new HIV infections in the United States are among young people aged 13-24 years, and 92% of these new infections are diagnosed in YMSM, making them one of the largest risk groups for HIV [1]. Men who have sex with men (MSM) of color are disproportionately impacted by the epidemic; in 2015, significantly more black MSM were diagnosed with HIV than white MSM, despite the relatively lower numbers of black MSM overall. HIV diagnoses among young Latino MSM have increased by 14% [1]. Importantly, it is estimated that compared with the general population, a higher proportion of YMSM (13% vs 44%, respectively) living with HIV do not know that they are infected [2,3] and, thus, will incur a delay seeking effective treatment, making it more likely that they will transmit HIV to others.

Overwhelming evidence demonstrates that routine testing, resulting in early identification, and therefore, early treatment, of HIV infection, and expanded use of pre-exposure prophylaxis (PrEP) would dramatically reduce the population burden of HIV as well as improve health outcomes for those who are infected [4-8]. However, HIV testing and use of PrEP among young adults is suboptimal. While at least biannual HIV testing is recommended for sexually active MSM, data suggest that approximately 60% of YMSM do not get even annual HIV tests [9]. Moreover, uptake of PrEP has remained low [10], particularly among young people. In a national Web-based survey of MSM, only 6% of those aged 18-24 years had ever used PrEP compared with 18% of those in the 30+ age group [11].

The normal developmental trajectory of adolescence and young adulthood involves behavioral experimenting, risk taking, and confronting a host of difficult choices with regard to identity formation [12]. These age-appropriate behaviors, beliefs of invincibility, and still-developing cognitive processes may play a role in increasing HIV risk taking behaviors and in placing a low priority on HIV testing and uptake of prevention strategies, particularly PrEP, which requires taking a daily pill to be effective [13].

The ongoing and growing HIV risk for YMSM highlights the need to reach younger individuals using developmentally appropriate, innovative methods and modalities. In addition to expanding access to effective prevention modalities, innovative methods to reach YMSM “where they are” must be developed. Smartphone use is nearly universal among youth in the United States [14]. Younger adults, racial and ethnic minorities, and socioeconomically disadvantaged populations have been identified as having high rates of smartphone use, reducing concerns of inequitable access to the technology [15]. The use of mobile phone apps is ubiquitous and is a common way in which youth interact, get information, and meet sex partners. Mobile apps offer unique opportunities for public health interventions, including efforts to increase HIV testing and PrEP uptake, particularly for YMSM, who may be open to receiving information in a familiar and discreet environment.

Although the popularity of mobile health apps is growing, there are limited data on the efficacy of app-based interventions to enhance HIV prevention and increase HIV testing among MSM, particularly YMSM. However, formative research suggests that Web-based, mobile, or social media outlets are acceptable and feasible means to increase the uptake of prevention services and HIV testing among MSM [16-20]. Informed by extensive formative research, Dr. Patrick Sullivan of Emory University (one of iTech’s Principal Investigators), together with app developers from Keymind, developed and tested an HIV testing promotion app for adult MSM (HealthMindr) [21]. Our initial formative research with YMSM [22] suggested interest in the basic functionalities of an HIV testing app like HealthMindr, but the youth felt that it would not be culturally and developmentally appropriate for their peers without further development. As a result, MyChoices incorporates youth feedback to realize an app that draws on the HIV testing functionality of HealthMindr but has been significantly redesigned in the following ways: (1) sexual health information presented in a simple, streamlined, and integrated fashion; (2) designed with attention to having a youth-friendly, social media style appeal; and (3) reduction of text by employing icons, graphics interchange formats (GIFs), and videos.

### Theoretical Framework for Intervention

**Figure 1:**
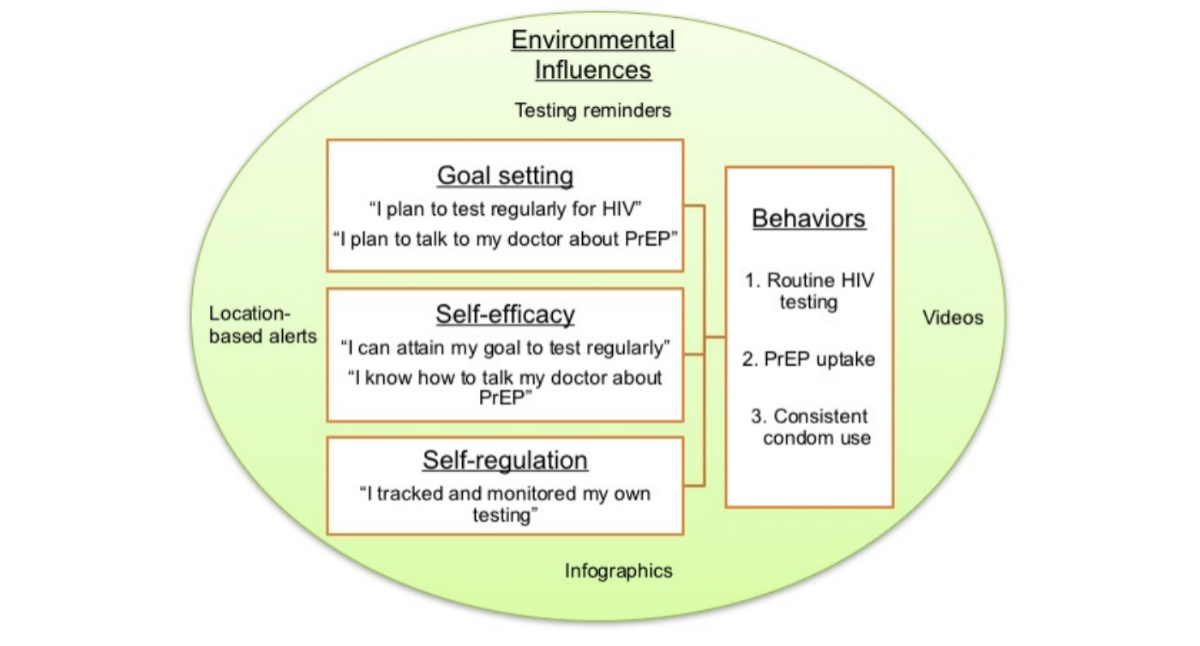
Theoretical model for the MyChoices app development. PrEP: pre-exposure prophylaxis.

The MyChoices app is informed by the social cognitive theory (SCT), which specifies goal setting, self-efficacy, and self-regulation as essential influences of health behavior [23,24]. SCT holds that cognition, behavior, and environmental influences interact with one another and reinforce one another to impact health behavior (Figure 1).

For example, self-regulatory functions (eg, self-monitoring one’s HIV testing through development of testing plans) and self-efficacy (eg, belief that one can attain the goal to test regularly) are enhanced by facilitative environmental conditions [23,24] such as reminder systems. MyChoices uses multimedia capabilities and environmental influences to support self-regulation and self-efficacy by enhancing the feeling of control over one’s ability to get tested regularly for HIV and use PrEP. Brief surveys about sexual risk and protective health behaviors within the app are used to assist users in tracking and self-monitoring their behaviors and creating a personalized HIV testing plan. Quizzes, videos, and infographics as well as “Help me Choose” (a quiz to help recommend a preferred setting for HIV testing), “Ordering” (a Web-based store to order free condoms, condom-compatible lubricant, and at-home HIV test kits), and geofencing (using global positioning system [GPS] to notify users when they are near a test site and due for testing) functions are used to maximize self-efficacy around HIV prevention and uptake of PrEP.

### Aims and Objectives

The aim of this paper is to describe the protocol for the refinement of an HIV prevention mobile app, beta testing it, and conducting a pilot randomized controlled trial (RCT) to examine the acceptability and feasibility of the MyChoices app for YMSM. We hypothesize that participants who are randomized to use the MyChoices app will report that the app is highly acceptable and will use the main functions of the app over the follow-up period. We also hypothesize that while not powered to detect significant differences, MyChoices users will report taking more HIV tests and increasing PrEP uptake compared with YMSM receiving the control condition.

## Methods

### Phase 1: App Refinement

To refine and enhance the MyChoices app for HIV testing and PrEP uptake among YMSM, we will conduct 4 theater testing groups with approximately 30 YMSM. Testing will take place at 2 iTech subject recruitment venues (SRVs; Boston, MA, and Bronx, NY), and groups will provide suggestions and feedback to improve app acceptability and feasibility as well as approaches to HIV prevention [25]. We aim to have 5-8 participants per group in order to balance the need to have an intimate enough group to share insights, yet large enough to ensure diversity of opinions [26,27]. Participants will be HIV-uninfected YMSM aged between 15 and 24 years who have not had a recent HIV test. For 15-17-year olds, participants must also self-report any anal sex with a male or transfemale partner. For 18-24-year olds, participants must self-report at least one of the following in the past 6 months:

1. ≥1 episode of condomless anal sex with an HIV-positive or unknown HIV status male or transfemale partner
2. Anal sex with ≥2 male or transfemale partners
3. Exchange of money, gifts, shelter, or drugs for anal sex with a male or transfemale partner
4. Sex with a male or transfemale partner and having had a sexually transmitted infection (STI)
5. Sexual partner of an HIV-positive man or transfemale with whom condoms were not consistently used

From our prior experience in recruiting YMSM, we found that it is necessary to include YMSM who engage in lower sexual risk (eg, anal sex with condoms) for the younger age group. Given that risk changes over time, we will still be enrolling an at-risk population who could benefit from the app and who should be testing for HIV regularly.

Prior to theater testing, participants will complete a brief demographic and behavioral questionnaire in order to contextualize the group data. During theater testing, participants will be asked to interact with the MyChoices app, and feedback will be elicited on the overall appearance and functionality of the app interface, appeal, and usability; ways to maximize acceptability (eg, update language, improve flow, etc); components they like and/or dislike; and areas for improvement. Guided by the SCT model, we will specifically obtain insight into the functionalities and content aimed at impacting self-regulation, self-efficacy, and environmental influences as they relate to HIV prevention.

Theater testing groups will last 60-90 minutes, and discussions will be audiorecorded and transcribed verbatim. Facilitators will also take notes and complete standardized debriefing forms immediately following the visit. These data will allow us to make final refinements to the app, as well as the intervention protocol and assessment battery, prior to initiation of the open technical evaluation and RCT pilot.

### Phase 2: Beta Testing

After refinement as described above, the MyChoices app will undergo beta testing with a small group of YMSM participants (up to 15 YMSM at 2 iTech SRVs: Boston, MA, and Bronx, NY) over a 2-month period. Using the same criteria as Phase 1, participants will be HIV-uninfected young cisgender MSM aged between 15 and 24 years who have not had a recent HIV test and self-report evidence of risk for acquiring HIV infection. For this phase, participants will also be required to own or lease an Android phone, as MyChoices will only be available on an Android platform during beta testing until it is coded by the same developers in iOS for the pilot RCT. This delay is meant to reduce the cost of recoding multiple versions of the edited app. Besides the slight native differences and system-specific limitations between the two platforms, the app was designed in such a way that the visual elements, capabilities, and interactions would be as similar as possible across both platforms.

All participants will be given brief instructions on the purpose of the MyChoices app and an overview of how to use it. Because the primary objective of the beta testing phase is to test instruments and procedures to be used in the pilot RCT, participants will complete the same assessments and processes to be used in the pilot RCT (see pilot RCT section for details). In brief, we will assess acceptability of the MyChoices app using the System Usability Scale (SUS) [28], and feasibility will be assessed using app analytics to determine whether the app was used and what functionalities were most likely and least likely to be opened. Participants will also be asked to provide feedback during Web-based exit interviews conducted by study staff using a Health Insurance Portability and Accountability Act-compliant videoconferencing software (VSee; VSee Lab, LLC, Sunnyvale, CA) technology. Feedback will be requested on functionality, technical performance, errors and software bugs encountered, overall experiences using the app, feedback for further refinement, and subjective impact of the app on HIV testing and PrEP uptake.

A rapid analysis of the data from the exit interviews will be conducted using the detailed notes taken on the debrief forms [29]. The study team will meet to discuss themes that emerged in the qualitative exit interviews, usage patterns, and acceptability ratings. We will triangulate the findings in order to refine the app, intervention protocol, and assessment tools prior to the pilot RCT [30].

### Phase 3: Pilot Randomized Controlled Trial

#### Study Design

**Figure 2:**
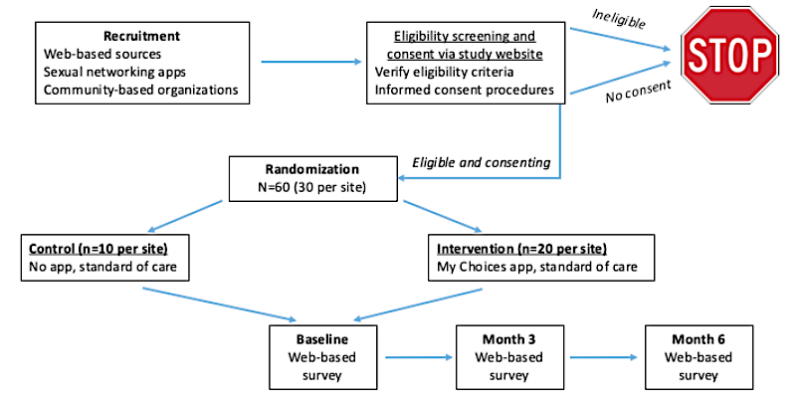
The MyChoices pilot randomized controlled trial schema.

A pilot RCT will be conducted at 2 iTech SRVs (Boston, MA, and Bronx, NY) to evaluate the feasibility and acceptability of the MyChoices app and examine the preliminary efficacy of the app in increasing HIV testing and PrEP uptake compared with a standard of care control group. This information will determine whether MyChoices moves forward to be tested in a full-scale efficacy trial planned with iTech. We will enroll 60 YMSM across 2 iTech SRVs who will be randomized to receive either the MyChoices app or standard of care. Participants will be followed for 6 months and will complete a Web-based assessment every 3 months (Figure 2).

#### Trial Registration, Ethics, Consent, and Institutional Board Approval

The research and ethics presented in this study have been reviewed and approved by the University of North Carolina at Chapel Hill Institutional Review Board (17‑0256). A Certificate of Confidentiality has been obtained from the National Institute of Child Health and Human Development, and a waiver of parental consent will be obtained for participants who are 15-17 years old. The study is also registered on ClinicalTrials.gov (NCT03179319). All participants will undergo screening in a private room at the clinical research site. If eligible (see below), the informed consent discussion will be conducted at this time. The informed consent documents will include detailed information on all study procedures and answer questions concerning the study and assent or consent process.

#### Participants

Study participants (N=60) will be assessed for eligibility by completing a brief screener. Inclusion criteria are identical to those in Phase 2 (beta testing), except that participants must own and be willing to download the app to a phone running either an Android or iOS platform. YMSM of color will be oversampled in order to reach a target enrollment of approximately 50% and to ensure sufficient representation of the population with the most need.

#### Recruitment

Active recruitment will be carried out by study staff at the SRVs by recruiting individuals from organizations and venues where YMSM attend. This may include community-based organizations for sexual and gender minority youth, youth Pride events, etc. Additionally, passive approaches for recruitment will include posting study information at these venues via flyers, posters, and palm cards describing the study. Web-based recruitment will be conducted by placing banner advertisements on popular Web-based social media outlets (eg, Facebook, Grindr, etc).

#### Randomization

Only participants who express interest in using MyChoices for HIV prevention, meet eligibility criteria, provide informed consent, and complete a baseline assessment will be eligible for randomization (see Figure 2). Randomization will be stratified by SRV [31] and will occur in a 2:1 ratio with 40 YMSM randomized to the experimental condition (20 at each SRV) and 20 randomized to the control condition (10 at each SRV). This allocation will allow us to efficiently gather additional data on app utilization. Randomization will be based on a pregenerated list created by the iTech Analytic Core statisticians, with random blocks of size 3 or 6, and will be accessed through a Web portal.

Men who are randomized to receive MyChoices will be given brief instructions on the purpose of the app, how to access it, and an overview of how to use it. Participants will be encouraged to explore all components of the app and use it routinely.

#### Incentives

Participants in the pilot RCT will receive US $50 compensation for the in-person screening or baseline assessment and US $25 compensation for each completed Web-based follow-up assessment.

#### Intervention

##### Standard of Care Condition

Following completion of the baseline assessment via computer-assisted self-interviewing (CASI), participants in both conditions will receive written prevention material including recommendations for HIV testing and referrals to local HIV testing sites and prevention services.

##### MyChoices Intervention Condition

**Figure 3:**
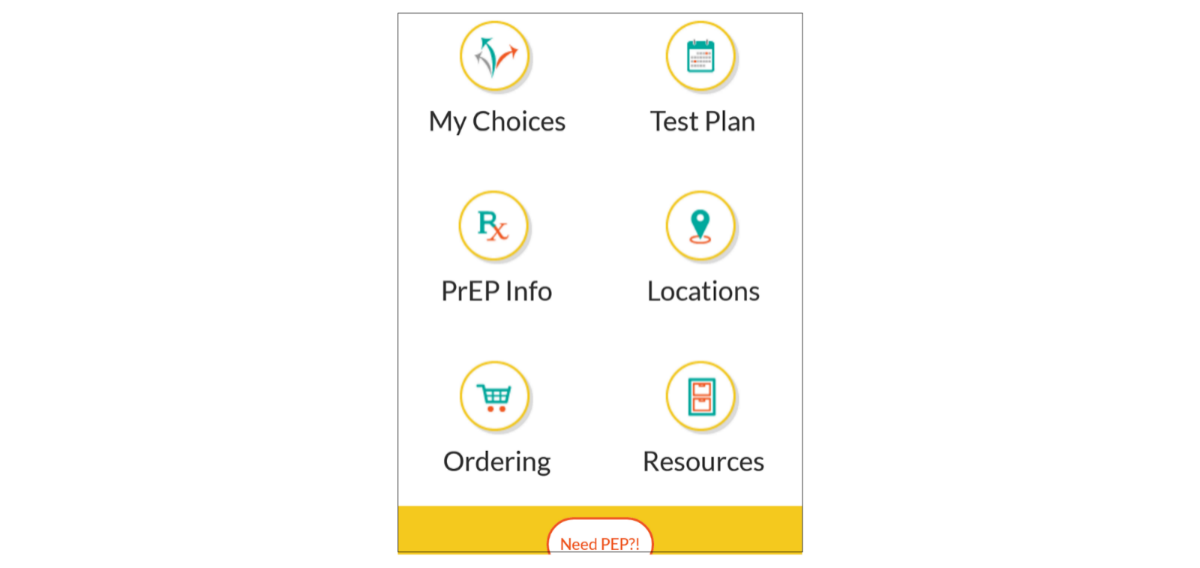
The MyChoices home screen.

The main functions of the app coincide with the core elements of the SCT of behavior change and are described below (see Figure 3).

###### Self-Regulation of HIV Risk

Brief surveys within the app are used to assess individual behavior patterns, particularly around sexual relationships. This information is used to customize the users’ app experience. For example, users who report having a main partner will be informed about couples counseling and users who report difficulties using condoms will be provided information about PrEP and links to HIV prevention services at local clinical sites. Users will be asked to complete these brief surveys monthly, which will allow recommended prevention activities to be updated based on recent behaviors. Users are also able to enter HIV and STI test results to keep track of past testing dates and results.

###### Self-Efficacy for HIV Testing and HIV Prevention

A number of components target self-efficacy by empowering YMSM to be confident that they can effectively manage engaging in HIV testing, PrEP uptake, and condom use. For example, quizzes, infographics, and GIFs that focus on the promotion of HIV prevention and regular HIV testing and that appeal to YMSM have been incorporated. Links to videos related to PrEP (eg, what is PrEP and talking to your doctor about PrEP), reasons for routine HIV testing, and the importance of engaging in care if one tests positive are included. The MyChoices app also allows users to order home-testing kits for HIV and STIs (syphilis and rectal and urethral gonorrhea and chlamydia) and different types of condoms and lubricants that can be shipped directly to them or to another more confidential location of their choosing via an arrangement with Amazon. Additionally, the app includes information on and links to testing sites and local PrEP clinics (eg, contact number, address, and testing hours) as determined using GPS technology, allowing men to locate clinics that are nearby their current location.

**Figure 4:**
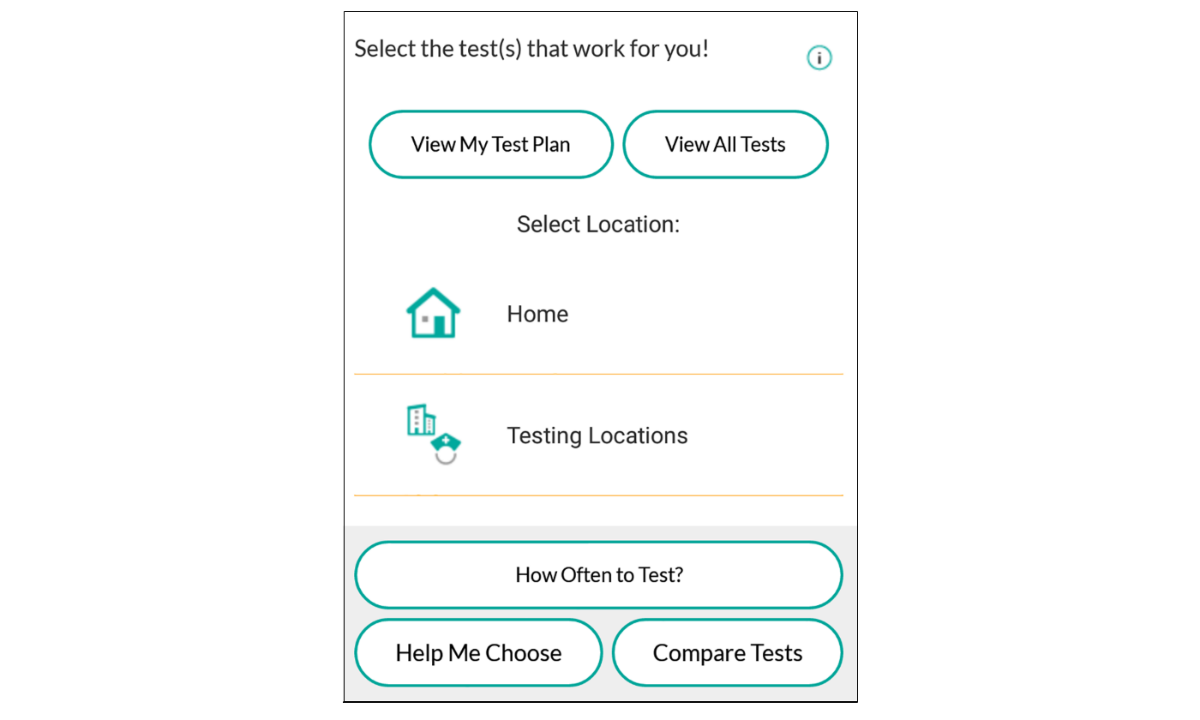
The MyChoices test plan.

###### Goal Setting and Environmental Influences

The MyChoices app allows users to create an individually tailored HIV testing plan by having them compare and choose different options (eg, antigen-antibody testing to identify very recent infection vs rapid testing at home or in a clinic for convenience and quick answers) and provides answers to questions about HIV transmission behaviors and testing history (Figure 4).

After an HIV testing plan is developed, users have the option to customize the timing and content of testing reminders (eg, users can select from a list of phrases or create their own reminder to ensure privacy). Geofencing technology provides users with notifications to test if in the vicinity of a testing location (based on their GPS location) during the testing timeframe.

#### Data Collection

Baseline assessments will be conducted at the enrolling clinical site using CASI, with all follow-up assessments at 3 and 6 months conducted off-site and through Web. At each major assessment, participants will complete a CASI-based assessment battery via a secure Web-based data entry system. Outcome domains and measures are described in Table 1.

**Table 1:** Outcomes and measures for MyChoices pilot randomized controlled trial.

Domain			Description or scale
**Primary outcomes**			
	App acceptability		System Usability Scale [28,32]
	App feasibility		Proportion using the app ≥1 time
**Secondary outcomes**			
	HIV testing		Proportion testing at least once during study
	PrEP^a^ uptake		Proportion of those with a behavioral indication for PrEP who are prescribed and utilize PrEP
**Social cognitive theory model constructs outcomes**			
	Self-regulation		Frequency of use of relevant app components, perceived HIV or STI^b^ risk [33]
	Self-efficacy		HIV testing self-efficacy [34], PrEP use self-efficacy [35], condom use self-efficacy [36]
	Goal setting		Frequency of use of HIV testing plan
	Environmental influences		Frequency of use of reminders, frequency of testing due to geofencing technology
**Covariates (based on ecosocial model)** [37]			
	**Individual**		
		Demographics, socioeconomic position	Age, race or ethnicity, student status, education, income, family structure, employment, insurance status
		Sexual behavior (# sex partners, condom use, partner selection)	Adapted from the AIDS Risk Behavior Assessment [38]
		Drug use behavior (ie, alcohol, cocaine, meth)	Alcohol, Smoking, and Substance Involvement Screening Test [39]
		Mental health (depression, anxiety)	Personal Health Questionnaire Depression 8-Item Scale [40], Generalized Anxiety Disorder 7-Item Scale [41], sexual minority stress [42]
		Trauma and abuse	Startle, Physically Upset, Anger, and Numbness Posttraumatic Stress Scale [43]
	**Social**		
		Social support	Patient-Reported Outcomes Measurement Information System [44]
		Peer norms for condom use	Questions regarding peer’s use and perceptions for condoms [45,46]
	**Structural**		
		SRV^c^ or city	Geographic location of study participant
		Incarceration history	Recent history and frequency
		Structural discrimination	External homophobia [42], racism [47]
		Stigma	HIV-related, PrEP-related
**Other covariates**			
	Mobile phone and technology use		Pew research technology use questionnaire [48,49]
	Mobile app use over the study period		Log in attempts, HIV testing and PrEP use, proportion complete HIV testing plans, proportion requesting HIV or STI home-test kits
	HIV Negative Cascade		HIV or STI testing history, PrEP awareness, barriers to PrEP uptake, PrEP adherence [50], barriers to PrEP use

^a^PrEP: pre-exposure prophylaxis.

^b^STI: sexually transmitted infection.

^c^SRV: subject recruitment venues.

##### Primary Outcome Measures

To measure acceptability of the MyChoices app, we will use the SUS, a validated 10-item measure that assesses subjective usability of a system, or, in this case, an app [28]. It is scored from 0 to 100, and a score of ≥50 indicates that the app is acceptable [32]. To determine feasibility, we will use app analytics to determine whether at least 60% of individuals randomized to the intervention condition opened the My Choices app at least once after their initial introduction to the app by research staff. We will also assess the proportion of participants who complete their HIV testing plan (regardless of planned testing intervals)—a primary function of the app.

##### Secondary Outcome Measures

While this pilot study is not powered to examine the efficacy of behavioral outcomes, at each major assessment, participants will be asked to self-report on HIV testing in the previous 3-month interval. We will also assess self-report accuracy by obtaining medical release for HIV test results. Additionally, at each assessment, participants will self-report whether, in the past 3 months, they made and attended a clinic appointment for PrEP initiation, whether they were prescribed PrEP, and whether they utilized PrEP.

##### Tertiary Outcome Measures

All measures are outlined in detail in Table 1. In brief, congruent with our theoretical model, we will assess the following: (1) Self-regulation: perceived HIV and STI risk and transmission knowledge; (2) Self-efficacy: HIV testing, PrEP use, and condom use self-efficacy; and (3) Environmental influences: perceived utility of reminders for HIV testing. Each of these variables will mirror the content of the app, and we will adapt validated scales when available [51]. We will also assess individual (eg, sexual behavior and mental health), social (eg, social support), and structural (eg, incarceration and stigma) covariates. Finally, among those randomized to the MyChoices condition, we will use app analytic data to assess the frequency and duration of app use, content and functionalities most and least utilized, and requests for HIV or STI testing kits, condoms, and lube.

### Statistical Analyses

The primary analyses will summarize acceptability (mean score on the SUS) and feasibility (participants utilizing the app at least once during follow-up) of the app, overall for the intervention arm, with asymptotic normal 95% CIs. Point estimates for mean acceptability ≥50 and for proportion accessing the app >0.60 will be considered the minimum criteria for acceptability and feasibility, consistent with industry standards [28,32].

The primary efficacy analysis will compare HIV testing (defined by the proportion that self-reports at least 1 HIV test result during follow-up) between the study arms at 3- and 6-month follow-ups. Group differences in self-reported PrEP-related appointments and documented HIV test results will also be examined at each time point. Moreover, group differences in measures related to the SCT model constructs (eg, HIV testing self-efficacy) will be assessed. The distribution of all variables will be assessed, and appropriate tests will be conducted (ie, parametric chi-square test vs nonparametric Fisher’s exact test).

All analyses will use two-tailed tests of significance, with significance at alpha=.05. We will follow an intent-to-treat approach [52], analyzing participants in the study arm to which they were assigned. We will examine the equivalence of random assignment to groups with regards to key baseline characteristics, including sociodemographics, prior HIV testing patterns, and sexual risk-related variables. In the event that randomization does not work to balance these characteristics, we will assess whether baseline differences may account for differences in outcomes.

## Results

Recruitment for the RCT began in October 2018, with study follow-up complete by June 2019.

## Discussion

Advances in mobile phone technologies have enabled YMSM to have immediate access to broad social and sexual networks. The proposed project responds to the increasingly widespread use of mobile technology in the United States. MyChoices is a theory-driven app that was developed by our interdisciplinary team of HIV clinicians, epidemiologists, behavioral scientists, and app developers based on feedback from a diverse sample of YMSM and will be refined and tested using scientifically rigorous research methods. Therefore, to our knowledge, MyChoices will be one of the first comprehensive, theory-driven HIV prevention apps designed specifically for YMSM. We anticipate that MyChoices will increase HIV testing and linkage to prevention services because it is developmentally appropriate and meets YMSM where they are, in an environment that is familiar and discrete.

Anticipated limitations of this protocol include the self-reported outcomes for HIV testing and PrEP uptake. We will explore obtaining more objective measures of HIV testing and PrEP uptake, including obtaining photographs of test results and PrEP prescriptions. Relatedly, by answering questions about their HIV testing history at follow-up assessments, self-reported outcomes may be influenced that could bias the results. However, the follow-up survey covers a number of topics, including mental health, stigma, and sexual behaviors, and the HIV testing questions are limited. As a result, we do not anticipate substantial misclassification. There is also a risk that participants in the intervention group may discuss with or even show the MyChoices app to participants in the standard of care group (eg, if friends or partners both enroll in the study). To reduce the risk of contamination, study staff will remind the participants at baseline not to discuss the app with peers who might be in the study.

If MyChoices demonstrates acceptability and feasibility in this pilot study, a multicity, 3-arm RCT of this app and another youth-optimized app (LYNX) versus standard of care will be conducted through iTech. If shown to be efficacious, the MyChoices app could be a scalable technology-based solution, with the ability to reach YMSM across the United States, while maintaining a “geographically individualized” feel with app content integrated with local prevention and testing activities.

## Abbreviations

CASI: computer-assisted self-interviewing
GPS: global positioning system
GIF: graphics interchange format
MSM: men who have sex with men
PrEP: pre-exposure prophylaxis
RCT: randomized controlled trial
SCT: social cognitive theory
SRV: subject recruitment venues
STI: sexually transmitted infection
SUS: System Usability Scale
YMSM: young men who have sex with men
